# Co-AMPpred for in silico-aided predictions of antimicrobial peptides by integrating composition-based features

**DOI:** 10.1186/s12859-021-04305-2

**Published:** 2021-07-30

**Authors:** Onkar Singh, Wen-Lian Hsu, Emily Chia-Yu Su

**Affiliations:** 1 Bioinformatics Program, Taiwan International Graduate Program, Institute of Information Science, Academia Sinica, Taipei, Taiwan; 2 Institute of Biomedical Informatics, National Yang Ming Chiao Tung University, Taipei, Taiwan; 3 Graduate Institute of Biomedical Informatics, College of Medical Science and Technology, Taipei Medical University, 250 Wu-Xing Street, Taipei, 11031 Taiwan; 4 Clinical Big Data Research Center, Taipei Medical University Hospital, Taipei, Taiwan

**Keywords:** Antimicrobial peptide, Amino acid composition, Composition-based feature, Machine learning

## Abstract

**Background:**

Antimicrobial peptides (AMPs) are oligopeptides that act as crucial components of innate immunity, naturally occur in all multicellular organisms, and are involved in the first line of defense function. Recent studies showed that AMPs perpetuate great potential that is not limited to antimicrobial activity. They are also crucial regulators of host immune responses that can modulate a wide range of activities, such as immune regulation, wound healing, and apoptosis. However, a microorganism's ability to adapt and to resist existing antibiotics triggered the scientific community to develop alternatives to conventional antibiotics. Therefore, to address this issue, we proposed Co-AMPpred, an in silico-aided AMP prediction method based on compositional features of amino acid residues to classify AMPs and non-AMPs.

**Results:**

In our study, we developed a prediction method that incorporates composition-based sequence and physicochemical features into various machine-learning algorithms. Then, the boruta feature-selection algorithm was used to identify discriminative biological features. Furthermore, we only used discriminative biological features to develop our model. Additionally, we performed a stratified tenfold cross-validation technique to validate the predictive performance of our AMP prediction model and evaluated on the independent holdout test dataset. A benchmark dataset was collected from previous studies to evaluate the predictive performance of our model.

**Conclusions:**

Experimental results show that combining composition-based and physicochemical features outperformed existing methods on both the benchmark training dataset and a reduced training dataset. Finally, our proposed method achieved 80.8% accuracies and 0.871 area under the receiver operating characteristic curve by evaluating on independent test set. Our code and datasets are available at https://github.com/onkarS23/CoAMPpred.

**Supplementary Information:**

The online version contains supplementary material available at 10.1186/s12859-021-04305-2.

## Background

### Antimicrobial peptides (AMPs)

In 1928, Alexander Fleming accidentally discovered the first commercialized antibiotic, “Penicillin, " that enormously changed the world of medicine [1]. Over the period, this finding was turned into a wonder drug that can miraculously cure bacterial infections in patients, and countless lives have been saved [2]. Since then, several antibiotics were discovered that contributed to revolutionizing the 20th-century healthcare system and achieved undeniable success in treating and deterring infectious diseases [3]. Unfortunately, however, unnecessary prescribing and overprescribing of antibiotics over the years lead to antibiotic resistance in microbes [4]. According to the World health organization (WHO) report, 0.7 million people die each year due to antibiotic-resistant disease, including 0.23 million deaths from multidrug-resistant tuberculosis, which is perhaps the major public health concern today.

Antibiotic resistance is defined as the ability of the pathogen to resist antibiotics to which they were first sensitive [5]. There are various mechanisms by which microorganisms gain resistance to antibiotics. First is by limiting the uptake of antibiotics by reducing their permeability, as in Gram-negative bacteria [6]. Compared to the peptidoglycan layer of Gram-positive bacteria, Gram-negative species have a lipopolysaccharide outer membrane, which is a superior permeability barrier for bacteria to keep drugs out [7]. Similarly some bacteria may also gain resistance to specific antibiotics by altering the hydrophobic properties of the outer membrane barrier [8]. Second is by modifying the drug targets as the antibiotics may target multiple components of the bacterial cell. Therefore, in response to the drug, bacteria also modify its components to enable resistance. For example, Gram-positive bacteria alter the structure and number of PBPs (Penicillin-binding proteins) against *β*-lactam drugs. PBPs are transpeptidase enzymes that help in cell wall biosynthesis by cross-linking peptidoglycans. An increase or decrease in the PBPs affects the drug binding to its target [6]. Third is by the inactivation of the drug through its modification by the bacterial enzyme. For example, *β*-lactamase can hydrolyze many *β*-lactam antibiotics like penicillin, cephalosporin, carbapenems, etc., making them ineffective [9]. Fourth way of bacterial resistance to antibiotics is by drug efflux. Exposure of the antibiotic activates bacterial pathways causing overexpression of transporter gene and efflux pumps that can pump out the antibiotics before reaching their target sites imparting resistance to the bacteria [10, 11]. Lastly, microbes also gain resistance by bypassing the effects of antibiotics by developing new cellular processes. For example, trimethoprim drug targets prokaryotic dihydrofolate reductase (DHFR) enzyme activity required for DNA synthesis more efficiently than eukaryotic DHFR. Nevertheless, *Staphylococcus aureus* bacteria gain resistance to the antibiotic by substituting amino acid in the chromosomally encoded DHFR or by horizontal transfer of plasmid encoding DHFR enzyme, which is not sensitive to inhibition [12].

As described above, resistance to an antibiotic is a major public health concern, and the development of new therapeutics alternatives is much needed. Antimicrobial peptides (AMPs) are promising potential candidates to serve as an alternative to antibiotics to counteract multidrug-resistant in microbes. AMPs are ancient conserved gene-encoded molecules that act as critical components of host innate immunity against invading pathogens. These oligopeptides naturally occur in multicellular organisms as the first line of defense against invading microbes [13]. These peptides exhibit a broad spectrum of antibacterial activities against gram-positive and gram-negative bacteria. AMPs consist of positively charged (cationic) residues (arginine and lysine) and a large portion (30–60%) of hydrophobic residues [14]. The basic properties (amphipathicity, cationic charge, and helical structure) of these residues permit them to interact and disturb membranes with negatively charged lipopolysaccharide membranes (outer membrane) or with the cytoplasmic membrane composed of lipoteichoic acids and peptidoglycan of gram-positive bacteria via ‘barrel-stave’, ‘carpet’, or toroidal pore mechanisms [15]. Moreover, AMPs also have antibacterial, antifungal, antiviral, and antiparasitic activities [16].

Since the first AMP was discovered, researchers have been inclined to understand the importance of amino acid residues in antimicrobial activity to design and yield better peptides [17]. Typically, amino acids were substituted to redesign peptides with increased positive charges and hydrophobic residues. However, several studies led to the discovery of distinct cationic host defense peptides (CHDPs) comprising magainins [18], cathelicidins [19], defensins [20], and cecropins [21]. These peptides have remarkably different structures and bioactivity profiles from conventional drugs [22]. Comprehensive work done in this field concluded that these bioactive peptides act as direct antimicrobial agents and are crucial regulators of the innate immune response. They can promote recruitment and accumulation of various immune cells at inflammatory sites, enhance phagocytosis, stimulate angiogenesis and induce wound repair [23]. Contrary to this, all conventional drug screening and design approaches require considerable patience and commitment, intensive effort, and atrocious costs with an ancillary workforce. Additionally, the experimental validation of a vast array of molecules for specific healing properties is comparatively challenging.

### Literature review

In the past two decades, numerous sequence-based in silico methods were reported to help develop novel candidate molecules. Generally, these prediction methods were based on exploring sequence-based and physiochemical-based properties of AMPs with machine-learning methods. Spänig et al. have recently presented a review introducing existing important encodings of amino acids and the efficient models for AMP classification [24]. Xiao et al*.* proposed a two-level prediction method based on pseudo amino acid compositions with a fuzzy K-nearest neighbour (FKNN) algorithm [25]. Mehar et al*.* developed a prediction method based on a support vector machine with compositional, physicochemical, and structural features of peptides [26]. Bahdra et al.proposed AmPEP, a random forest (RF) classifier-based prediction model in which distribution patterns of amino acid properties were used as input to develop a highly accurate prediction model [27]. In 2018, Veltri et al*.* developed the first deep-learning method with primary sequence composition; for that, they proposed a neural network model with convolutional and recurrent layers. The datasets used in the study were 1,778 AMPs and 1,778 non-AMPs, respectively, downloaded from the APD vr.3 and UniProt databases. Their proposed method's overall performance showed an accuracy of 91.0% and an area under the receiver operating characteristics curve (AUROC) of 0.964 [28]. Lin et al*.* developed a MAMP-Pred prediction model to address the multilevel problem with the PS-RF and LC-RF classifiers’ help. They obtained their dataset from APD database with 2,618 AMPs, while the 4,371 non-AMP sequences were obtained from UniProt database. The overall performance of this model had 85.6% accuracy [29]. Yan et al*.* proposed another convolutional neural network (CNN)-based deep learning prediction model, DEEP-AmPEP30, with the help of PseKRAAC, with reduced amino acid compositions to predict short AMPs. The dataset used in their study consisted of comparably shorter (5 ~ 30 amino acid residues) peptide sequences than previous studies. DEEP-AmPEP30 outperformed the existing methods with a similar dataset with an overall accuracy of 77.1% and AUROC of 0.851 [30]. ACEP, a deep neural network (DNN)-based deep learning method that used a convolutional layer and LSTM layer to generate feature tensors of the dataset comprising 3,556 peptide sequences, was divided into three parts: 1,424 for training, 708 for tuning, and 1,424 for testing; they achieved an accuracy of 93% with the test dataset [31]. All existing methods [28, 29, 31] were developed based on AMP collections without considering sequence lengths. This observation was first reported by Yan et al*.* [32]*,* tested all existing methods on short AMPs with lengths of 5 ~ 33 amino acid residues and found that the prediction accuracy ranged 65% ~ 73%, which was far worse than previously reported accuracies of 90% ~ 95%. The significant variation displayed by the existing models indicated that large sequences might not contain the optimal compositions for antimicrobial activity, and large sequences with 80 ~ 255 amino acid residues may contain sequence segments that do not depict antimicrobial activity. However, AMPs are short-length peptides ranging from 5 ~ 30 amino acid residues. Accordingly, it is reasonable to use a maximum sequence cut of 30 amino acid residues with optimal sequence compositions to enhance a model’s effectiveness. We further elucidated a hypothesis proposed by the previous study. We developed our model based on several encoding schemes of amino acids compositional and physicochemical properties for a short-length AMP dataset.

### Challenges in AMP predictions

Despite many attractive advantages of AMPs over conventional drugs including lesser development of resistance against AMPs [33], inhibition of biofilm formation [34], and the modulation of host immune response [35], many fail to reach the market because of their low stability, shorter half-lives, and challenges with oral delivery, immunotoxicity, cytotoxicity, and most importantly, higher manufacturing costs. Several chemical strategies were established to address these problems but ended unsuccessfully with higher manufacturing costs and limited response rates. Thus, instead of conventional approaches for predicting AMPs, researchers have recently been inclined towards in silico approaches to elucidate the mechanisms of direct killing of pathogens and assist the pharmaceutical industry in developing novel therapeutics.

### Specific aims of this study

The broad spectra of antimicrobial activities of AMPs with lower rates of resistance development make AMPs promising candidates for developing novel broad-spectrum antibiotics. This study proposes a prediction method in which composition-based sequence and physicochemical features are computed for short-length AMPs incorporated into several machine-learning algorithms. First, stratified tenfold cross validation was performed on the training dataset to test and evaluated multiple times. Second, boruta feature selection algorithm was used to identify discriminative features. The constructed classifier was used to evaluate the model on the holdout test dataset. The proposed AMP prediction method can further help to develop more-potent antimicrobial agents.

## Results

In the current study, we have used various approaches to classify AMPs over non-AMPs. Here we have elaborated all the analysis done in this study, such as AMPs sequence preference and compositional analysis, model development on the state-of-art dataset, and the reduced dataset generated by applying CD-HIT at various sequence identity thresholds. The detailed information is mentioned in the method section.

### Sequence preference analysis and compositional analysis

In this study, we visually investigated differences in amino acid residues between positive and negative dataset based on positional information of charged and hydrophobic residues within the primary sequence of the AMP peptides with the help of a two-sample logo (TSL). The height of the peptide logo was scaled (*t*-test by *p* < 0.05) for statistical significance. To further examine amino acid residues' preferences at the N and C termini, we selected the greatest length (i.e., 30 amino acids) of AMPs and non-AMPs. Since, the sequence length of all the peptides differs, therefore, we used padding (-) to make the peptide lengths equal. Thus, the first 15 amino acid residues represented the N-terminal, and the last 15 residues represented the C-terminal. Notably, the most significant amino acid represents the relative abundance in the sequences.

**Fig. 1:**
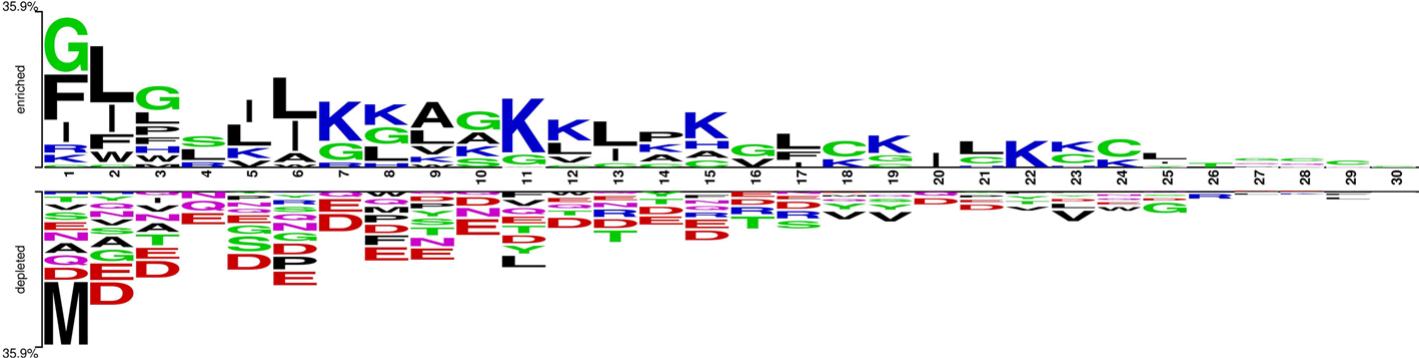
Two-sample logo shows the preference of positively charged and hydrophobic residues in antimicrobial peptides (AMPs) and non-AMPs at different positions. The first 15 positions represent the N-terminus of peptides, and the last 15 positions represent the C-terminus of peptides

Upon examination of the preference analysis based on charge and hydrophobicity, we found that in Fig. 1, positively charged residue, i.e., lysine (K), frequently occurred at the 7th, 8th, 11th, 12th, and 15th positions in the N-terminal, as well as the 19th, 22nd, and 23rd positions in C-terminal of AMPs. On the other hand, in non-AMPs negatively charged residues (aspartic acid and glutamic acid) were frequently present at the 2nd, 3rd, 4th, 5th, 6th, 7th, 8th, 9th, 10th, 12th, 14th, and 15th in N-terminal positions, as well as the 20th and 21st positions in the C-terminal. These data indicate that in AMPs, lysine is preferred in N-terminals among other cationic residues, while in non-AMPs, negatively charged residues are abundant in the N-terminus and dominated almost at every position. Similarly, the preference of hydrophobic amino acid residues such as leucine (L), isoleucine (I), and alanine (A) in AMPs were frequent at 2nd, 4th, 5th, 6th, 9th, and 13th positions of the N-terminal. Whereas at C-terminus, 17th, 20th, 21st, and 25th positions of L and I residues were frequent. Conversely, if we look at non-AMPs, hydrophobic residues occur only at 11th position in the N-terminal and at 18th, 19th, and 23rd positions in the C-terminal.

### Compositional analysis of AMP datasets

**Fig. 2:**
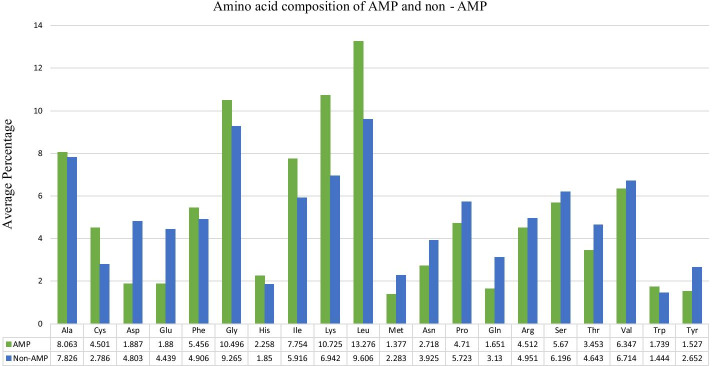
Average percentages of amino acid compositions (AACs) in antimicrobial peptides (AMPs) and non-AMPs

Proteins are combinations of small molecules naturally present in polypeptides, known as proteinogenic or natural amino acids. Various organisms can diversify these amino acids into hormones, enzymes, antibodies, antibiotics, and many more with discrete biological activities. AMPs are cationic (positively charged) and amphiphilic (hydrophilic and hydrophobic) in nature [36]. With this knowledge, we analyzed AACs of both positive and negative AMP datasets. Average compositions of AMPs and non-AMPs are shown in Fig. 2. The average composition of positive residues such as lysine (K) and histidine (H) and hydrophobic residues such as alanine (A), isoleucine (I), leucine (L), proline (P), and tryptophan (W) in AMPs were higher than those in non-AMPs. Besides, negatively charged residues such as aspartic acid (D) and glutamic acid (E) were more abundant in non-AMPs than in AMPs.

### Machine-learning prediction models on the DEEP-AmPEP30 dataset

**Fig. 3:**
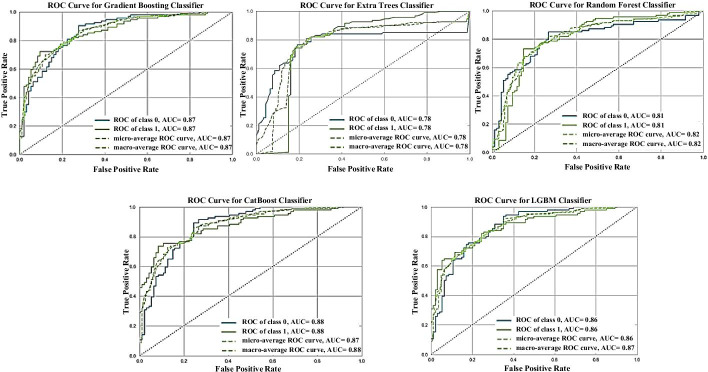
The area under the receiver operating characteristic (AUROC) curve shows the models' performance developed using selected features on the independent test dataset

In our study, we used various machine-learning algorithms with the help of PyCaret, a python library. At first, we collected the dataset from a previous study [32]. Furthermore, we computed a vast array of feature descriptors (1,400 feature descriptors) for a given dataset. Then, we performed stratified tenfold cross-validation (CV) on the DEEP-AmPEP30 training dataset. Next, we used boruta feature-selection algorithm with a cutoff of 0.9 to select essential features, i.e., 70 feature descriptors. Then, we implemented several machine-learning algorithms on the selected features. To finalize the best model among the classifiers, GBC achieved the optimum performance with an AUROC of 0.814, an accuracy of 75.0%, and MCC of 0.504 on the training dataset, and an AUROC of 0.871 with an accuracy of 80.8%, and MCC of 0.606 on the test dataset with an AUCPR of 0.89. We listed the other classifier’s predictive performance on the benchmark DEEP-AmPEP30 training and independent test datasets in Table 1, and the AUROC values are given in Fig. 3.

**Table 1:** Performances of machine-learning models on the benchmark training and independent test datasets. Values shown are mean ± SD for the training dataset

Algorithm	Dataset	Accuracy	AUROC	Recall	Precision	Kappa	MCC
GBC	Training	75.0% ± 0.038	0.816 ± 0.035	77.4% ± 0.082	73.9% ± 0.033	0.500 ± 0.0755	0**.**504 ± 0.075
	Test	**80.3%**	**0.873**	**79.7%**	**80.6%**	**0.606**	**0.606**
CatBoost	Training	74.4% ± 0.055	0.815 ± 0.045	75.3% ± 0.107	73.9% ± 0.045	0.488 ± 0.110	0.492 ± 0.109
	Test	78.7%	0.879	78.7%	78.7%	0.574	0.574
LGBM	Training	73.8% ± 0.060	0.810 ± 0.052	73.3% ± 0.124	73.8% ± 0.039	0.476 ± 0.102	0.479 ± 0.099
	Test	77.6%	0.868	78.7%	77.0%	0.553	0.553
ETC	Training	74.3% ± 0.055	0.794 ± 0.066	75.0% ± 0.097	73.9% ± 0.049	0.487 ± 0.109	0.491 ± 0.108
	Test	77.6%	0.776	77.6%	77.6%	0.553	0.553
RF	Training	74.1% ± 0.044	0.798 ± 0.052	75.5% ± 0.101	73.1% ± 0.039	0.482 ± 0.088	0.487 ± 0.086
	Test	78.1%	0.811	78.7%	77.8%	0.563	0.563

The given data in bold font indicates the top performance of the model on the test dataset

GBC, gradient boosting classifier; LGBM, light gradient boosting machine; ETC, extra trees classifier; RF, random forest; AUROC, area under the receiver operating characteristics curve; MCC, Mathew's correlation coefficient; SD, standard deviation

**Fig. 4:**
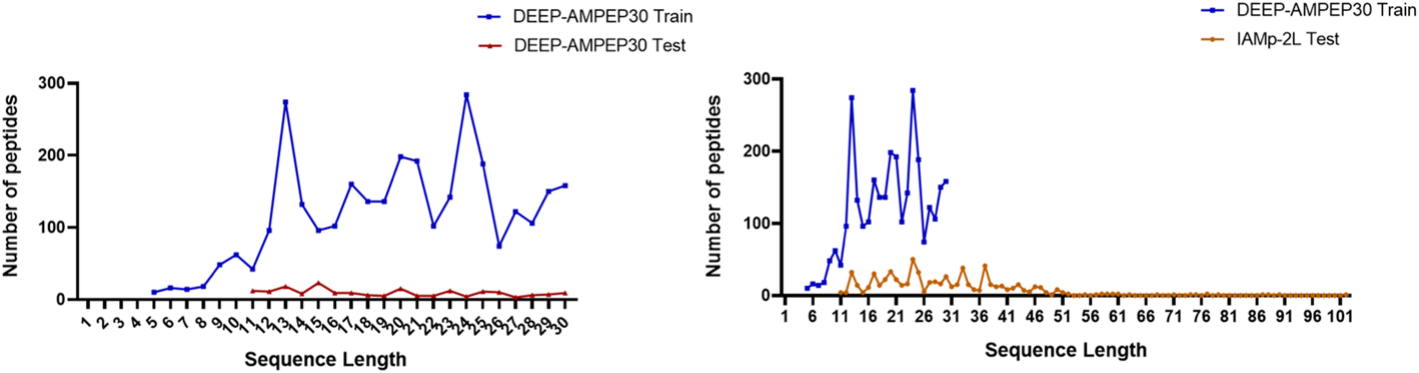
Sequence length distribution between training and test dataset. a) Sequence length distribution between DEEP-AMP30 training and independent test dataset. b) Sequence length distribution between DEEP-AMP30 training and iAMP-2L independent test dataset

Additionally**,** to evaluate the robustness of our model on the different independent test set. We retrieved the dataset used in the previous study IAMp-2L [25]. Initially, the test dataset contains 920 AMPs and 920 non-AMPs sequences. Then, we further processed the data by applying CD-HIT at a 90% sequence identity threshold to remove redundant sequences and finally, we obtained 674 AMPs and 630 non-AMPs. Our best model GBC achieved the AUROC of 0.951 with an accuracy of 88.3%. Contrastingly, we observed improved performance on the iAMp-2L test dataset compared to the DEEP-AmPEP30 independent test dataset. This improved performance is mainly affected by peptide sequence length distribution in both (DEEP-AmPEP30 and iAMp-2L) independent test sets and the benchmark training dataset. The differences in sequence length distribution are shown in Fig. 4. The performance of our models on the iAMp-2L test set is shown in Additional file 1.

### Machine-learning model predictions on the reduced training datasets

CD-HIT is indeed the state-of-art method that is widely used in biological studies. We used CD-HIT to examine the benchmark dataset to minimize redundant sequences and address the overfitting problem. We used three different (i.e., 90%, 80%, and 70%) sequence identity thresholds to create three different training datasets. All these training datasets were used to train the model. Furthermore, we computed all feature descriptors listed in Table 2. We performed stratified tenfold cross-validation on the training set to fit and evaluate models’ multiple times. Based on the wrapper algorithm, we used boruta feature-selection method with a selection threshold of 0.9 to select the most important biological features, i.e., 171, 98, and 96 feature descriptors. With the obtained feature set, we used several machine-learning algorithms to predict AMPs on the holdout set. We listed all prediction performances in Additional files 2–4.

**Table 2:** List of all descriptors along with their abbreviations and numbers of features

Feature type	Descriptor	Abbreviation	No. of features
Simple composition	Amino acid composition	AAC	20
	Dipeptide composition	DPC	400
	Atom-type composition	ATC	5
	Bond-type composition	BTC	4
Physicochemical properties	Amino acid index	AAI	553
	Physicochemical property	PCP	30
Distribution & repeats	Distance distribution of repeats	DDR	20
	Residue repeat information	RRI	20
	Property repeat index	PRI	24
Shannon entropy	Shannon entropy of a residue	SER	20
	Shannon entropy of properties	SEP	25
	Shannon-entropy of a protein	SE	1
Miscellaneous	Amphiphilic pseudo amino acid composition	APAAC	23
	Pseudo amino acid composition	PAAC	21
	Composition enhanced transition and distribution	CeTD	189
	Quasi-sequence order	QSO	42
	Sequence order coupling number	SOC	2

## Discussion

This study tried to understand the importance of the PCPs of AMPs and their sequence-based amino acid compositions. The dataset had significant impact on machine-learning tasks. Hence, we collected training and benchmark test datasets from a previous study [32]. First, we developed our model on the same dataset for a comparative analysis with the state-of-the-art method and created other reduced datasets with CD-HIT at 90%, 80%, and 70% sequence identity thresholds from the training dataset. Second, we used available software packages and webservers to compute 1,400 feature descriptors from the peptide sequences.

### Evaluation of the top 10 selected features

**Fig. 5:**
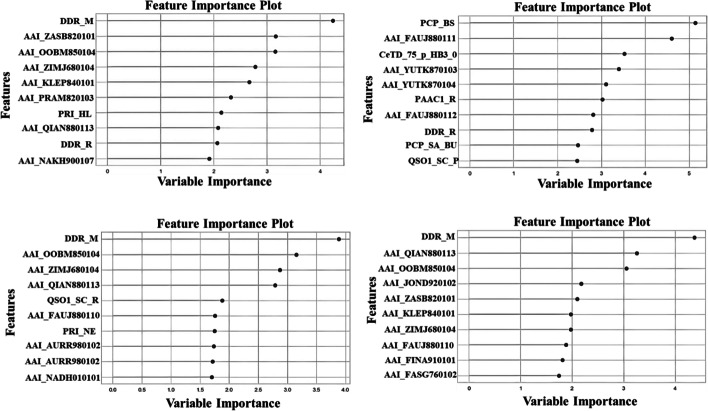
Top 10 feature importance plot for benchmark DEEP-AMP30 training and reduced training datasets at 90%, 80%, and 70% sequence identity thresholds

For real-world machine-learning problems, data representation often uses several features. However, few of them may be relevant to the target variable. In such cases, feature selection is crucial to accelerate the learning process and improve prediction performance [37]. Therefore, we have applied feature selection on our dataset that was initially created by Bhadra et al*.,* in their study AmPEP, by retrieving naturally occurring and experimentally validated AMP sequences from three major databases namely CAMPR3 [38], APD3 [39], and LAMP [40]. Since, there is not enough evidence reported for experimentally validated non-AMPS in literature. Therefore, the author followed data preparation procedure undertaken by other studies to create negative dataset [25, 41]. Furthermore, all sequences retrieved from UniProt were processed by removing sequences that were annotated as AMP, membrane, toxic, secretory, defensive, antibiotic, anticancer, antiviral, and antifungal. Subsequently, the subset of the AmPEP dataset was used in the state of art method Deep-AmPEP30. Similarly, we used benchmark training dataset from the state of art method and generated three additional training datasets by applying CD-HIT with the cutoff of 90%, 80%, and 70% to remove highly similar sequences within the Deep-AmPEP30 training set. Later, we computed vast array of features from available webservers and standalone packages. To identify discriminative biological features for predicting AMPs, the boruta feature-selection algorithm was used to select optimal features among other feature descriptors. This algorithm works on an all-relevant variable selection method, where the boruta algorithm attempts to curate the subset of features from the dataset to all-relevant stopping points to identify relevant features for a given classification task [42]. As a result, we respectively obtained 70, 171, 98, and 96 feature descriptors for the benchmark training dataset and the three other reduced training datasets. Finally, we presented the top 10 selected feature importance plots in Fig. 5.

Based on the structure activity relationship (SAR) studies of antimicrobial peptides, various parameters have been shown to influence the specificities and biological activity of peptides. These parameters are secondary structure, charge, hydrophobicity, and amphipathicity [43]. In general, AMPs are classified into four major structural categories such as helical peptides, β-strand/sheet peptides, mixed helical/sheet peptides and extended non-helical/sheet peptides [44]. However, amphipathic, α-helical conformation is assumed to be the important class of AMPs as they permit efficient interaction with the lipid bilayer [45]. Most AMPs are known to carry net positive charge ranging from + 2 to + 9 and target negatively charged bacterial membranes through electrostatic interactions [46, 47]. Moreover, as we increase the net positive charge up to + 9 antimicrobial activity gradually increases. However, if the charge increases beyond + 9 the antimicrobial activity decreases [47]. Hydrophobicity is essential factor for interaction with membranes, and as it is believed that cytoplasmic membrane is the main target of AMPs and hydrophobicity is the crucial parameter for their biological activity. Usually AMPs contain approximately 50% hydrophobic residues [48]. Similar to charge, various studies have revealed that increasing the hydrophobicity, at optimal hydrophobicity window, can increase the antimicrobial activity [49]. The importance of charge, structure and hydrophobicity is not limited as we discussed above, but these three factors also jointly form an amphipathic structure, that too is of greater importance. Usually all AMPs form some kind of amphipathic structure which is crucial factor for potent anti-microbial activity of AMPs [50].These physicochemical property of AMPs allows peptides to attack membrane by interacting with the hydrophobic-hydrophilic character of the lipids. The quantitative measure of amphipathicity is hydrophobic moment (μH) that is the total of amino acid two dimensional vectorial hydrophobicity’s [51]. Several studies reported that hydrophobic moment is strongly correlated with antimicrobial activity, as an increase in hydrophobic moment will increase the disruption of bacterial membrane and hemolytic activity [49].

Interestingly, we found in our study that the selected features fell into parameters which modulate AMP activities and specificities. These includes, structure, charge, hydrophobicity, and amphipathicity that were equally important in our datasets, for example top features like distance distribution for residues methionine (DDR_M) and arginine (DDR_R) imparts two major characteristics, hydrophobicity and positive charge, respectively. Hydrophobicity is an essential feature for AMP-membrane interactions [52] and cationic residues help AMPs to direct them to negatively charged bacterial membranes via electrostatic attraction [53]. Therefore, balance between these two characteristics is crucial for selective antimicrobial activity. Physicochemical features generated from AAI includes alpha-helix weight at position 6 (QIAN880113), hydrophobicity (ZIMJ680104), net charge (KLEP840101), dependence of the partition coefficient on the ionic strength (ZAS820101), non-bonded energy per atom (OOBM850104), activation of Gibbs energy (YUTK870103), normalized positional frequency at helix termini C4’ (AURR98012), and the hydropathy scale based on the self-information value in two-state model (NADH010101).

Using stringent criteria to reduce redundant sequences from the state-of-the-art dataset, we observed that our machine learning models’ performances gradually decreased in the training dataset and increased in the test dataset. In Sect. 3.4, we discussed three different training datasets created from the benchmark DEEP-AmPEP30 training dataset by reducing peptide sequences at various sequence identity threshold (CD-HIT, 90%, 80%, and 70%). By applying CD-HIT with a 90% sequence identity threshold, we obtained 2136 peptide sequences (1076 AMPs and 1060 non-AMPs). GBC achieved 92.9% accuracy on this training data and 70.2% accuracy on the test dataset (Additional file 2). Similarly, we applied CD-HIT at 80% sequence identity threshold where we get a total of 1957 peptides (946 AMPs and 1011 non-AMPs). GBC achieved 80.6% accuracy on this training dataset and 76.6% accuracy on the test dataset (Additional file 3). After applying CD-HIT at 70%, the total number of sequences obtained was significantly reduced as compared to the original dataset, comprising 1697 peptides (787 AMPS and 910 non-AMPs). Overall performance on CD-HIT 70% training dataset obtained by GBC is 79.9% accuracy, and 78.1% accuracy on the test dataset (Additional file 4). Therefore, we hypothesized that to develop a more accurate and reliable method; ideally, one should use more stringent redundant sequence-reduction criteria to train the model and evaluate it on an experimentally verified independent test dataset.

### Positional preferences and composition analysis of AMPs

The widespread class of AMPs is cationic amphipathic with an alpha-helical domain [54]. These AMPs have two distinct features, i.e., a net positive charge and an amphipathic character, with a nonpolar face and a polar/charged face [55]. Similarly, the top selected features emphasized the characteristics of AMPs. In addition, we used TSL and compositional analytics to analyze preferential positions of amino acid residues in AMPs and non-AMPs. Our positional analysis based on charge revealed that the positively charged lysine (K) residue often occurred in AMPs, while negatively charged aspartic acid and glutamic acid residues were abundantly present in non-AMPs. In addition, a preference study based on hydrophobic residues suggested that hydrophobic residues such as leucine (L), isoleucine (I), and alanine (A) were favored in AMPs and not in non-AMPs.

### Performance comparison with state-of-the-art methods

Using the benchmark dataset and the reduced training dataset at various sequence identity thresholds, we compared our Co-AMPpred (composition-based antimicrobial peptide prediction) method with six state-of-the-art general AMP prediction methods. As shown in Table 3, our Co-AMPpred and CO-AMPpred70 classifiers outperformed all available AMP prediction methods and short-length AMP prediction models on most performance matrices. Co-AMPpred attained the best performance with 80.3% accuracy, an AUROC of 0.871, and MCC of 0.606 on the benchmark dataset. Moreover, CO-AMPpred70 achieved better performance than the state-of-the-art methods with an accuracy of 78.6%, an AUROC of 0.861, and MCC of 0.554. In this study, we used various approaches to examine the performance of our model. We first developed our model on the benchmark Co-AMPpred dataset and attained the best performance among other classifiers developed in this study. Then, to further evaluate our model performance, we created other dataset by applying CD-HIT at various sequence identity thresholds to reduce redundant sequences from the benchmark training dataset. Among all classifiers developed on reduced training datasets (i.e., Co-AMPpred70, Co-AMPpred80, and Co-AMPpred90), Co-AMPpred70 attained the best performance and outperformed the existing state-of-the-art methods.

**Table 3:** Performance comparison with existing methods on the benchmark test dataset

Method	Acc	AUROC	AUCPR	Kappa	Sen	Spe	MCC	References
iAMP-2L	65.4%	–	–	0.318	82.9%	47.9%	0.329	Xiao et al. [25]
iAMPpred	70.7%	–	–	0.415	80.8%	60.6%	0.424	Meher et al. [26]
AmPEP	68.0%	0.751	0.686	0.362	93.6%	42.5%	0.421	Bhadra et al. [27]
AMP Scanner DNN	73.4%	0.806	0.777	0.468	80.8%	65.9%	0.473	Veltri et al. [28]
RF-AmPEP30	77.1%	0.854	0.868	0.543	77.6%	76.6%	0.542	Yan et al. [32]
Deep-AmPEP30	77.1%	0.853	0.853	0.543	76.6%	77.7%	0.543	Yan et al. [32]
Co-AMPpred	**80.8%**	**0.871**	**0.890**	**0.606**	79.7%	**81.9%**	**0.606**	This study
Co-AMPpred70	78.6%	0.861	0.860	0.553	80.9%	74.5%	0.554	This study
Co-AMPpred80	76.6%	0.851	0.840	0.532	78.7%	74.5%	0.532	This study
Co-AMPpred90	70.2%	0.843	0.860	0.404	**89.4%**	51.1%	0.438	This study

The given data in bold font indicates the top performance of the model on the test dataset

Acc., accuracy; AUROC, area under the receiver operating characteristics curve; AUCPR, area under the precision-recall curve; Sen., sensitivity; Spe., specificity; MCC, Matthew's correlation coefficient; SD, standard deviation

### Limitations of the study

In our study, we developed a prediction method to identify AMPs and non-AMPs. The dataset we used comprised AMPs from different sources to develop our classification model. Tentatively, one should develop host-specific methods for predicting AMPs. We will shortly try to develop a host-specific AMP classification model with perfect size data to develop a precise and more reliable method. This study exploits several compositional and physicochemical-based features to develop the best possible models in the current situation.

## Conclusions

AMPs are evolutionary conserved molecules which act as the first defense line in all multicellular organisms. AMPs have different mechanisms to disrupt bacterial membranes. However, all these mechanisms are dependent on various factors such as physicochemical properties, amino acid sequences, secondary structures, charges, and amphipathic properties. AMPs can demonstrate a broad spectrum of activities to modulate immune responses and demonstrate antiviral, antifungal, antibacterial, and even anticancer activities [56]. Increasing resistance of microbes against conventional antibiotics motivates researchers to develop new therapeutic alternatives such as AMPs. In the past two decades, several in silico-based approaches were developed. However, in this study, we used various encoding schemes of an amino acid, compositional and physicochemical properties to develop our prediction model. As a result, the top selected informative features yield better performance and outperformed the-state-of-art-method.

Our findings also indicate that our classification task's top selected features reproduce the parameters that modulate AMPs' activities and specificities, such as structure, charge, hydrophobicity, and amphipathicity. Along with selected feature importance values, we further investigated the position preference and composition analysis of the AMPs/non-AMPs to understand the importance of amino acid compositions in AMPs. Finally, our investigation revealed that selected features imparted distinct characteristics of the amino acid residues available in AMPs. Although our positional preference and compositional analysis corresponded well with other biological studies [57], further insights should be validated experimentally in the future.

The state-of-the-art method inspired us to develop a model with short sequences of AMPs (5 ~ 30 residues). We also addressed the bias caused by redundant sequences within training data and developed a model based on reduced training datasets. Experimental results showed that the combining composition-based and physicochemical features outperformed existing methods on both the benchmark training dataset and the reduced training dataset at a 70% sequence identity threshold. However, we further observed that minimizing redundant sequences at various sequence identity thresholds affected the machine-learning prediction performances: the more stringent the criteria, the better was the prediction performance of the models on the independent test dataset.

## Material and methods

### Dataset preparation and pre-processing

We used an initial training dataset consisting of 1,529 AMP and 1,529 non-AMP sequences originally compiled in Deep-AmPEP30 [32]. The study aims to predict short anti-microbial peptides. Nevertheless, the test dataset used in the previous study was constructed without considering the peptide length. To address this issue, we constructed an independent dataset from the benchmark dataset reported in the recent publication [58]. Sequences that are 5–30 amino acids in length were taken as positive samples, whereas negative samples were selected randomly to generate a balanced test dataset by following the procedure reported in the state-of-the-art method. Furthermore, we checked whether or not the benchmark dataset contains highly similar sequences (> 90%) to either the training dataset of our method or existing AMP methods with which we made comparisons in our study. Then, CD-HIT was applied with an 80% cutoff to remove highly similar sequences within the dataset to reduce redundancy and avoid bias. Finally, the constructed independent set contains 94 AMPs and 94 non-AMPs.

To deal with the overfitting problem of the prediction model, we then performed a major pre-processing step. We first used CD-HIT [59, 60] to decrease sequence redundancy within the training data with maximum sequence identities of 90%, 80%, and 70%. After checking for any redundant peptides, a new training dataset was developed to train our model. However, the more stringent the criterion, steadier the performance like a 30% or 40% sequence identity cutoff we noted. Despite this, the sequence length of the reduced dataset was < 30 amino acid residues. If we applied a stringent criterion of < 70%, the number of available AMPs was significantly reduced, and we were unable to retrieve datasets employed by this state-of-the-art method. We describe all datasets with various sequence identity thresholds in Table 4.

**Table 4:** Benchmark datasets used for the antimicrobial peptide (AMP) prediction

Dataset	Training dataset		Test dataset	
	AMPs	Non-AMPs	AMPs	Non-AMPs
Benchmark datasets	1529	1529	94	94
CD-HIT (90%)	1076	1060	94	94
CD-HIT (80%)	946	1011	94	94
CD-HIT (70%)	787	910	94	94

### Feature extraction

Feature representation plays a crucial role in the accuracy prediction by machine-learning models. Sequence transformation is essential to obtain a numerical representation of amino acids before using them as input for machine-learning models. Various approaches have been reported to encode amino acid sequences into numerical vectors, which have been rigorously used in biomedical classification. However, there is no precise guideline published that allows researchers to use specific encodings for a biomedical classification task. Moreover, in a recent publication, Spänig et al. attempted to investigate the performance of various encoding schemes on previously published datasets. Their performance results indicate that none of the encodings are superior across all biomedical domains. Despite this, some encodings often perform better than others, thus reducing the initial encoding selection considerably [61]. It is evident that amino acids are the building blocks of peptides and proteins, and each of 20 amino acids maintains unique and different properties. The composition of amino acids with their unique properties can influence protein’s structural and functional diversification and characteristics. This study aimed to develop a prediction model by employing numerous features of protein and peptide sequences.

### Composition and physicochemical feature descriptors

Composition-based features and each amino acid residue's physicochemical properties are widely used in computational biology [62]. In the current study, we used available webservers and standalone packages to generate a broad spectrum of feature-encoding schemes derived from protein and peptide sequences [63, 64]. Composition-based features were subcategorized into five different modules. First, a simple composition included the amino acid composition (AAC), dipeptide composition (DPC), atom-type composition (ATC), and bond-type composition (BTC). The AAC represents the occurrence frequency of each amino acid in query peptides [65]. Similarly, the DPC calculates the amino acid pair frequency in query peptide sequences [66]. The second module represents physicochemical properties (PCPs) and amino acid index (AAI) of residues. This feature represents the overall sum of all PCPs and AAI residue values of discrete types. The third is a repeat and distribution module, which comprises three feature schemes of distance distribution of repeats (DDR), residue repeat information (RRI), and property repeat information (PRI). Fourth is the Shannon entropy module, which also consists of three feature descriptors, i.e., Shannon entropy of a residue (SER), Shannon entropy of properties (SEP), and Shannon-entropy of protein (SE) to measure the complexity at the protein and residue levels. Finally, the fifth module of composition-based features was a collection of several feature schemes. For example, the amphiphilic amino acid composition (APAAC) and pseudo amino acid composition (PAAC) are somewhat like the AAC and contain more information on discrete correlation factors. This additional information gives more insight into the hydrophobic and hydrophilic distribution patterns of peptide chains [67, 68]. Autocorrelation descriptors are used to compute the distribution of amino acid properties and sequences [69]. Composition-enhanced transition and distribution (CeTD) compute the overall composition, enhanced transition, and distribution (CTD) of amino acid attributes, such as hydrophobicity, normalized Van der Waal volume, polarity, polarizability, charge, secondary structure, and solvent accessibility of protein sequences [70]. Quasi-sequence order (QSO) and the sequence order coupling number (SOC) descriptors can be used to represent the distribution patterns of PCPs along the peptide sequence [71]. We describe the complete list of all descriptors along with feature numbers in Table 2.

### Machine-learning algorithms

In the present study, we used various machine-learning algorithms to develop a classification model for AMPs and non-AMPs. To deploy several machine-learning models together, we used PyCaret, an open-source, low code machine-learning library in python [72]. This python library includes 15 different machine-learning algorithms, including CatBoost classifier, gradient boosting classifier (GBC), extra trees classifier (ETC), extreme gradient boosting (XGB), light gradient boosting machine (LGBM), random forest (RF), ada boost classifier (ABC), logistic regression (LR), SVM-linear kernel, naive Bayes (NB), decision tree (DT), ridge classifier, K-nearest neighbor classifier (KNN), quadratic discriminant analysis (QDA), and linear discriminant analysis (LDA). We briefly describe information about the top five algorithms used for prediction. The CatBoost classifier is a newly developed machine learning algorithm based on gradient boosting [73]. GBCs are used for regression and classification problems. This model's strength is that it creates weak prediction models and merges them to produce the best prediction model [74]. The ETC is an ensemble machine-learning algorithm that creates many randomized decision trees using a training dataset and combines average prediction accuracies of all decision trees to improve the prediction accuracy [75]. Similarly, we used another supervised machine-learning method, the RF classifier, an ensemble learning method that creates a random decision tree from the training set and uses majority voting to identify the final output [76]. Light gradient boosting, a tree-based learning algorithm, grows vertically and can easily handle a large dataset with low memory consumption [77].

### Evaluation measures

Model assessment becomes crucial when the nature of predictions needs to be measured. We used a training set to build up or train the predictive model and a test set to test a classifier's performance. Although receiver operating characteristic (ROC) curves are the best choice for comparing models, we also considered other scalar metrics that are still popular among the machine learning community, such as recall/sensitivity (Sen.) in Eq. (1) and the specificity (Spe.) in Eq. (2), to measure how well a classifier detects AMPs and non-AMPs in the dataset. Precision, as shown in Eq. (3), defines the proportion of positively predicted AMPs that are true real positives. Accuracy (Acc.) in Eq. (4) is the summation of true positives and true negatives divided by the total number of the data. The area under the ROC curve (AUROC) [78] and the area under the precision-recall curve (AUCPR) [79]. Equation (5) states Matthew’s correlation coefficient (MCC), which is also used to measure the quality of our binary classification task, and Eq. (6) defines the kappa statistic [80].

1 $$Sen. = Recall = \frac{TP}{{TP + FN}}$$

2 $$Spe. = \frac{TN}{{TN + FP}}$$

3 $$Precision = \frac{TP}{{TP + FP}}$$

4 $$Acc. = \frac{TP + TN}{{TP + TN + FP + FN}}$$

5 $$MCC = \frac{TP \times TN - FP \times FN}{{\sqrt {\left( {TP + FP} \right)\left( {TP + FN} \right)\left( {TN + FP} \right)\left( {TN + FN} \right)} }}$$

where TP is the number of true positives, TN is the number of true negatives, FP is the number of false positives, and FN is the number of false negatives. The ROC curve is used to assess the performance during parameter selection; AUROC is the most appropriate performance measure, as it is non-parametric and threshold dependent. In the ROC curve, the true positive rate (sensitivity) is plotted as a function of the false positive rate (1—specificity) for different parameter cutoff points. The AUCPR plots the positive predictive value against the true positive rate. The MCC measures the quality of a binary classification task. Kappa statistics measure inter-rater reliability, where *p*_*0*_ is the overall accuracy of the model as shown in Eq. (7), and *p*_*e*_ is the measure of agreements between the model prediction and the actual class values expected by chance in Eq. (8) [80].

6 $$Kappa = \frac{po - pe}{{1 - pe}}$$

7 $$p_{0} = \frac{{{\text{TP}} + {\text{TN}}}}{{{\text{TP}} + {\text{FN}} + {\text{TN}} + {\text{FP}}}}$$

8 $$p_{e} = \frac{{\left( {{\text{TP}} + {\text{FN}}} \right) \times \left( {{\text{TP}} + {\text{FP}}} \right) \times \left( {{\text{TN}} + {\text{FN}}} \right) \times \left( {{\text{TN}} + {\text{FP}}} \right)}}{{({\text{TP}} + {\text{FN}} + {\text{TN}} + {\text{FP}})^{2} }}$$

We used a training dataset for internal validation, where models were trained and tested using a stratified10-fold cross-validation method. The constructed classifier was later used to evaluate our model on the holdout test dataset.

### System architecture

**Fig. 6:**
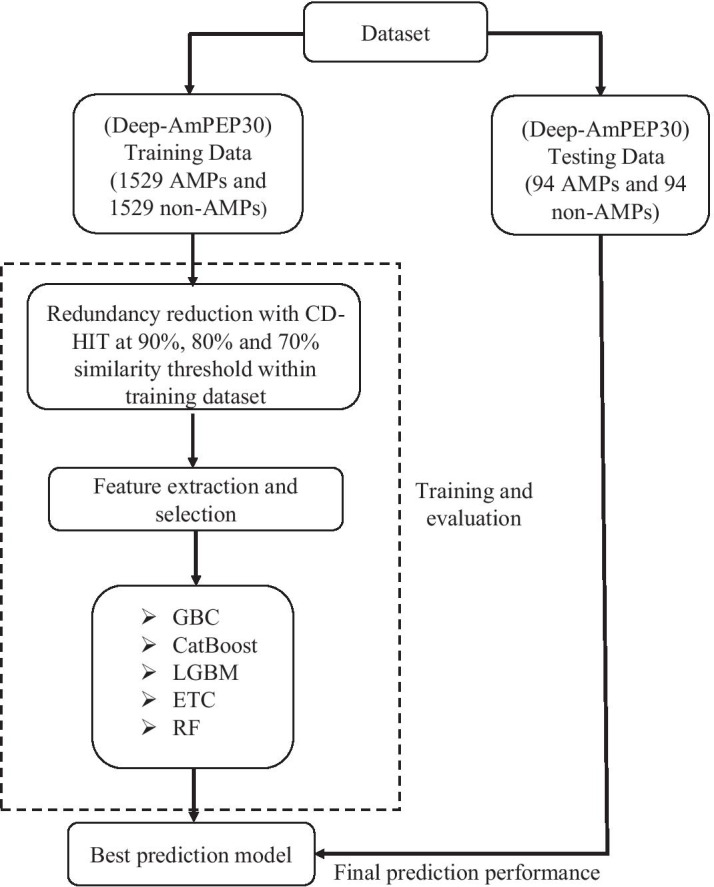
The systematic architecture of the proposed method, Co-AMPpred, includes collecting the dataset, removing redundant sequence at 90%, 80%, and 70% sequence identity thresholds, feature generation and selection, machine-learning algorithms, and evaluation process

The system architecture of our proposed method for predicting AMPs is shown in Fig. 6. The analytical workflow involved various steps, including collecting AMPs for input, feature extraction, feature selection, machine-learning algorithms, and prediction results. First, we downloaded oligopeptides using the existing state-of-the-art method. Along with the benchmark dataset, we created three additional datasets by applying CD-HIT with sequence identity thresholds of 90%, 80%, and 70% to reduce redundant sequences. Further, sequence-based compositional features and PCPs were extracted and encoded with the aid of various available webservers and standalone packages [63, 64] for both training and test dataset. Then, we performed a stratified tenfold CV on the training dataset. Boruta feature-selection algorithm was incorporated to select only discriminative biological features to build an optimal model that was later evaluated on the holdout set [42]. Finally, we examined all the models, and the execution of the prediction model was illustrated. Our system architecture represents the systematic procedures followed in this study. The name of our proposed method is Co-AMPpred (composition-based antimicrobial peptide prediction).

## Supplementary Information

**Additional file 1**. Performances of machine learning-based models using DEEP-AMP30 training dataset and IAMP-2L independent test dataset. Values shown are mean ± SD for the training dataset.

**Additional file 2**. Performances of machine learning-based models using 171 selected features on the reduced training (CD_HIT 90%) and independent test dataset. Values shown are mean ± SD for the training dataset.

**Additional file 3**. Performances of machine learning-based models using 171 selected features on the reduced training (CD_HIT 80%) and independent test dataset. Values shown are mean ± SD for the training dataset.

**Additional file 4**. Performances of machine learning-based models using 171 selected features on the reduced training (CD_HIT 70%) and independent test dataset. Values shown are mean ± SD for the training dataset.

## Data Availability

All data generated or analyzed during this study are included in the supplementary information. The codes and dataset are publicly available at https://github.com/onkarS23/CoAMPpred.

## Abbreviations

AAC: Amino acid composition
AAI: Amino acid index
ABC: Ada boost classifier
Acc.: Accuracy
AMP: Antimicrobial peptide
APAAC: Amphiphilic pseudo amino acid composition
APD: Antimicrobial peptide database
ATC: Atom-type composition
AUCPR: Area under the precision-recall curve
AUROC: Area under the receiver operating characteristic curve
BTC: Bond-type composition
CeTD: Composition enhanced transition and distribution
CHDP: Cationic host defense peptide
DDR: Distance distribution of repeats
DPC: Dipeptide composition
ETC: Extra trees classifier
FKNN: Fuzzy k-nearest neighbor
FN: False negative
FP: False positive
GBC: Gradient boosting classifier
KNN: K-nearest neighbor
LDA: Linear discriminant analysis
LGBM: Light gradient boosting machine
LR: Logistic regression
MCC: Matthew’s correlation coefficient
PAAC: Pseudo amino acid composition
PCP: Physicochemical property
PRI: Property repeats index
PseKRAAC: Pseudo k-tuple reduced amino acid composition
QDA: Quadratic discriminant analysis
QSO: Quasi-sequence order
RRI: Residue repeat information
SE: Shannon-entropy of protein
SEP: Shannon entropy of properties
SER: Shannon entropy of a residue
SOC: Sequence order coupling number
RF: Random forest
Sen: Sensitivity
Spe: Specificity
SVM: Support vector machine
TN: True negative
TP: True positive
TSL: Two-sample logo
XGB: Extreme gradient boosting
