# Supplementary material for: Co-AMPpred for in silico-aided predictions of antimicrobial peptides by integrating composition-based features
Source: BMC Bioinformatics. 2021 Jul 30;22:389. doi: 10.1186/s12859-021-04305-2 (PMC8325260; doi:10.1186/s12859-021-04305-2)
Supplement: Supplementary file 1 — Additional file 1. Performances of machine learning-based models using DEEP-AMP30 training dataset and IAMP-2L independent test dataset. Values shown are mean ± SD for the training dataset. [file 12859_2021_4305_MOESM1_ESM.docx]

| **Algorithm** | **Dataset** | **Accuracy** | **AUROC** | **RECALL** | **Precision** | **KAPPA** | **MCC** |
| --- | --- | --- | --- | --- | --- | --- | --- |
| GBC | Training | 0.7551 ± 0.0429 | 0.8145 ± 0.0437 | 0.7821 ± 0.1009 | 0.7439 ± 0.0379 | 0.5102 ± 0.0858 | 0.5161 ± 0.0834 |
|  | Test | 0.8839 | 0.9513 | 0.8539 | 0.9153 | 0.7681 | 0.7700 |
| CatBoost | Training | 0.7495 ± 0.0457 | 0.8113 ± 0.0478 | 0.7873 ± 0.0954 | 0.7335 ± 0.0401 | 0.4991 ± 0.0914 | 0.5048 ± 0.0899 |
|  | Test | 0.8978 | 0.9703 | 0.8465 | 0.9498 | 0.7961 | 0.8011 |
| ETC | Training | 0.7351 ± 0.0472 | 0.7981 ± 0.0515 | 0.7768 ± 0.0853 | 0.7177 ± 0.0452 | 0.4702 ± 0.0943 | 0.4755 ± 0.0963 |
|  | Test | 0.8678 | 0.9642 | 0.7690 | 0.9681 | 0.7371 | 0.7541 |
| RF | Training | 0.7397 ± 0.0336 | 0.8007 ± 0.0490 | 0.7938 ± 0.0995 | 0.7182 ± 0.0298 | 0.4794 ± 0.0672 | 0.4885 ± 0.0679 |
|  | Test | 0.9001 | 0.9716 | 0.8450 | 0.9562 | 0.8007 | 0.8065 |
| LGBM | Training | 0.7381 ± 0.0452 | 0.7987 ± 0.0530 | 0.7631 ± 0.0992 | 0.7285 ± 0.0413 | 0.4762 ± 0.0904 | 0.4815 ± 0.0888 |
|  | Test | 0.8809 | 0.9601 | 0.8331 | 0.9286 | 0.7623 | 0.7666 |

**Additional file 1.** Performances of machine learning-based models using DEEP-AMP30 training dataset and iAMP-2L test datasets. Values shown are mean ± SD

Acc., accuracy; AUROC, area under the receiver operating characteristics curve; MCC, Matthew's correlation coefficient; GBC, gradient boosting classifier; LGBM, light gradient boosting machine; ETC, extra trees classifier; RF, random forest; SD, standard deviation.
