# Supplementary material for: Co-AMPpred for in silico-aided predictions of antimicrobial peptides by integrating composition-based features
Source: BMC Bioinformatics. 2021 Jul 30;22:389. doi: 10.1186/s12859-021-04305-2 (PMC8325260; doi:10.1186/s12859-021-04305-2)
Supplement: Supplementary file 2 — Additional file 2. Performances of machine learning-based models using 171 selected features on the reduced training (CD_HIT 90%) and independent test dataset. Values shown are mean ± SD for the training dataset. [file 12859_2021_4305_MOESM2_ESM.docx]

**Additional file 2**. Performances of machine learning-based models using 171 selected features on the reduced training (CD-HIT 90% and independent test datasets. Values shown are mean ± SD

| Algorithm | Dataset | Acc.% | AUROC | Recall % | Precision % | Kappa | MCC |
| --- | --- | --- | --- | --- | --- | --- | --- |
| GBC | Training | 92.9% ± 0.022 | 0.979 ± 0.010 | 92.0% ± 0.037 | 94.0% ± 0.028 | 0.859 ± 0.044 | 0.861 ± 0.044 |
|  | Test | 70.2% | 0.843 | 89.4% | 64.6% | 0.404 | 0.438 |
| CatBoost | Training | 96.8% ± 0.015 | 0.996 ± 0.004 | 96.4% ± 0.026 | 97.2% ± 0.015 | 0.935 ± 0.031 | 0.936 ± 0.030 |
|  | Test | 64.4% | 0.841 | 95.7% | 59.2% | 0.298 | 0.378 |
| LGBM | Training | 94.6% ± 0.018 | 0.989 ± 0.007 | 92.8% ± 0.034 | 96.4% ± 0.022 | 0.892 ± 0.037 | 0.893 ± 0.036 |
|  | Test | 71.3% | 0.829 | 91.5% | 65.2% | 0.426 | 0.465 |
| ETC | Training | 92.9% ± 0.022 | 0.979 ± 0.011 | 90.1% ± 0.043 | 95.7% ± 0.025 | 0.859 ± 0.045 | 0.862 ± 0.043 |
|  | Test | 70.2% | 0.803 | 81.9% | 66.4% | 0.404 | 0.416 |
| RF | Training | 92.2% ± 0.022 | 0.974 ± 0.010 | 89.7% ± 0.036 | 94.6% ± 0.022 | 0.844 ± 0.045 | 0.846 ± 0.044 |
|  | Test | 71.3% | 0.755 | 84.0% | 66.9% | 0.426 | 0.440 |

Acc., accuracy; AUROC, area under the receiver operating characteristics curve; MCC, Matthew's correlation coefficient; GBC, gradient boosting classifier; LGBM, light gradient boosting machine; ETC, extra trees classifier; RF, random forest; SD, standard deviation.
