# Supplementary material for: Co-AMPpred for in silico-aided predictions of antimicrobial peptides by integrating composition-based features
Source: BMC Bioinformatics. 2021 Jul 30;22:389. doi: 10.1186/s12859-021-04305-2 (PMC8325260; doi:10.1186/s12859-021-04305-2)
Supplement: Supplementary file 3 — Additional file 3. Performances of machine learning-based models using 171 selected features on the reduced training (CD_HIT 80%) and independent test dataset. Values shown are mean ± SD for the training dataset. [file 12859_2021_4305_MOESM3_ESM.docx]

**Additional file 3**. Performances of machine learning-based models using 171 selected features on the reduced training ± CD-HIT 80% and independent test datasets. Values shown are mean ± SD

| **Algorithm** | **Dataset** | **Acc %** | **AUROC** | **Recall %** | **Precision %** | **Kappa** | **MCC** |
| --- | --- | --- | --- | --- | --- | --- | --- |
| GBC | Training | 80.6% ± 0.054 | 0.875 ± 0.065 | 77.9% ± 0.135 | 81.2% ± 0.032 | 0.610 ± 0.111 | 0.617 ± 0.099 |
|  | Test | 76.6% | 0.851 | 78.7% | 75.5% | 0.532 | 0.532 |
| CatBoost | Training | 81.4% ± 0.055 | 0.884 ± 0.056 | 78.7% ± 0.128 | 82.2% ± 0.038 | 0.627 ± 0.113 | 0.633 ± 0.104 |
|  | Test | 76.6% | 0.846 | 75.5% | 77.2% | 0.532 | 0.532 |
| LGBM | Training | 81.8% ± 0.056 | 0.884 ± 0.063 | 79.1% ± 0.135 | 82.4% ± 0.0376 | 0.634 ± 0.088 | 0.639 ± 0.115 |
|  | Test | 76.6% | 0.847 | 75.5% | 77.2% | 0.532 | 0.532 |
| ETC | Training | 80.1% ± 0.056 | 0.879 ± 0.060 | 75.9% ± 0.123 | 81.5% ± 0.039 | 0.599 ± 0.115 | 0.605 ± 0.109 |
|  | Test | 75.5% | 0.780 | 75.5% | 75.5% | 0.511 | 0.511 |
| RF | Training | 80.2% ± 0.057 | 0.873 ± 0.060 | 77.4% ± 0.125 | 80.8% ± 0.042 | 0.603 ± 0.083 | 0.609 ± 0.110 |
|  | Test | 78.2% | 0.820 | 78.7% | 77.9% | 0.564 | 0.564 |

Acc., accuracy; AUROC, area under the receiver operating characteristics curve; MCC, Matthew's correlation coefficient; GBC, gradient boosting classifier; LGBM, light gradient boosting machine; ETC, extra trees classifier; RF, random forest; SD, standard deviation.
