# Supplementary material for: Co-AMPpred for in silico-aided predictions of antimicrobial peptides by integrating composition-based features
Source: BMC Bioinformatics. 2021 Jul 30;22:389. doi: 10.1186/s12859-021-04305-2 (PMC8325260; doi:10.1186/s12859-021-04305-2)
Supplement: Supplementary file 4 — Additional file 4. Performances of machine learning-based models using 171 selected features on the reduced training (CD_HIT 70%) and independent test dataset. Values shown are mean ± SD for the training dataset. [file 12859_2021_4305_MOESM4_ESM.docx]

**Additional file 4.** Performances of machine learning-based models using 171 selected features on the reduced training CD-HIT 70% and independent test datasets. Values shown are mean ± SD

| **Algorithm** | **Dataset** | **Acc. %** | **AUROC** | **Recall %** | **Precision %** | **Kappa** | **MCC** |
| --- | --- | --- | --- | --- | --- | --- | --- |
| GBC | Training | 79.9% ± 0.062 | 0.870 ± 0.067 | 77.9% ± 0.106 | 78.3% ± 0.069 | 0.777 ± 0.127 | 0.595 ± 0.126 |
|  | Test | 78.1% | 0.857 | 81.9% | 76.2% | 0.789 | 0.564 |
| CatBoost | Training | 80.1% ± 0.053 | 0.874 ± 0.069 | 77.0% ± 0.122 | 79.3% ± 0.052 | 0.777 ±0.112 | 0.599 ± 0.107 |
|  | Test | 78.6% | 0.861 | 80.8% | 76.0% | 0.784 | 0.553 |
| LGBM | Training | 80.9% ± 0.055 | 0.879 ± 0.064 | 77.3% ± 0.121 | 80.8% ± 0.052 | 0.785 ± 0.114 | 0.616 ± 0.110 |
|  | Test | 77.1% | 0.855 | 78.7% | 76.3% | 0.775 | 0.543 |
| ETC | Training | 80.2% ± 0.058 | 0.873 ± 0.067 | 74.8% ± 0.124 | 80.8% ± 0.052 | 0.772 ±0.123 | 0.599 ± 0.118 |
|  | Test | 77.1% | 0.786 | 77.6% | 76.8% | 0.773 | 0.543 |
| RF | Training | 79.7% ± 0.617 | 0.865 ± 0.069 | 75.5% ± 0.119 | 79.5% ± 0.058 | 0.768 ±0.128 | 0.589 ± 0.125 |
|  | Test | 78.7% | 0.813 | 78.7% | 78.7% | 0.787 | 0.575 |

Acc., accuracy; AUROC, area under the receiver operating characteristics curve; MCC, Matthew's correlation coefficient; GBC, gradient boosting classifier; LGBM, light gradient boosting machine; ETC, extra trees classifier; RF, random forest; SD, standard deviation.

±
